# Identification of novel small RNAs in extracellular vesicles produced by *Actinobacillus pleuropneumoniae*

**DOI:** 10.3389/fmicb.2023.1291930

**Published:** 2023-11-23

**Authors:** Giarlã Cunha da Silva, Jéssica Nogueira Rosa, Patrícia Pereira Fontes, Alex Gazolla de Castro, Éverton De Almeida Alves Barbosa, Wellington Ronildo Clarindo, Hilário Cuquetto Mantovani, Yanwen Li, Janine Thérèse Bossé, Paul Richard Langford, Denise Mara Soares Bazzolli

**Affiliations:** ^1^Laboratório de Genética Molecular de Bactérias, Departamento de Microbiologia, Instituto de Biotecnologia Aplicada à Agropecuária (BIOAGRO), Universidade Federal de Viçosa, Viçosa, Brazil; ^2^Departamento de Microbiologia, Universidade Federal de Viçosa, Viçosa, Minas Gerais, Brazil; ^3^Departamento de Bioquímica e Biologia Molecular, Universidade Federal de Viçosa, Viçosa, Minas Gerais, Brazil; ^4^Departamento de Biologia Geral, Universidade Federal de Viçosa, Viçosa, Minas Gerais, Brazil; ^5^Section of Paediatric Infectious Disease, Department of Infectious Disease, Imperial College London, London, United Kingdom

**Keywords:** extracellular vesicles, Actinobacillus pleuropneumoniae, small RNAs, pathogenicity, virulence

## Abstract

Extracellular vesicle (EV) production by bacteria is an important mechanism for microbial communication and host-pathogen interaction. EVs of some bacterial species have been reported to contain nucleic acids. However, the role of small RNAs (sRNAs) packaged in EVs is poorly understood. Here, we report on the RNA cargo of EVs produced by the pig pathogen *Actinobacillus pleuropneumoniae*, the causal agent of porcine pleuropneumonia, a disease which causes substantial economic losses to the swine industry worldwide. The EVs produced by aerobically and anaerobically grown bacteria were only slightly different in size and distribution. Total cell and outer membrane protein profiles and lipid composition of *A. pleuropneumoniae* whole cell extracts and EVs were similar, although EVs contained rough lipopolysaccharide compared to the smooth form in whole cells. Approximately 50% of *Galleria mellonella* larvae died after the injection of EVs. RNAseq, RT-PCR, protection from nuclease degradation, and database searching identified previously described and 13 novel *A. pleuropneumoniae* sRNAs in EVs, some of which were enriched compared to whole cell content. We conclude that *A. pleuropneumoniae* EVs contain sRNAs, including those known to be involved in virulence, and some with homologs in other *Pasteurellaceae* and/or non-*Pasteurellaceae*. Further work will establish whether the novel sRNAs in *A. pleuropneumoniae* EVs play any role in pathogenesis.

## 1 Introduction

Extracellular vesicle (EV) production is a natural process documented in the three domains of life (Deatherage and Cookson, 2012; Gill et al., 2019). In general, bacterial EVs have a circular structure with sizes ranging between 20 and 400 nm (Toyofuku et al., 2019). Gram-negative bacterial EVs are predominantly composed of lipopolysaccharide (LPS) and proteins, but can also contain toxins, nucleic acids and other molecules (Gill et al., 2019). A plethora of functions have been assigned to EVs, including: cell-to-cell communication; mechanism for horizontal gene transfer (vesiduction), and participation in predatory mechanisms; biofilm formation; stress responses; antimicrobial resistance; delivery of toxins; and in secondary metabolism (Schwechheimer and Kuehn, 2015; Gill et al., 2019; Soler and Forterre, 2020). However, much remains to be elucidated, especially their contribution to microbial community and host-pathogen interactions. Due to their ability to generate an immune response, EVs have been used as vaccines (Micoli and Mac Lennan, 2020), e.g., to prevent *Neisseria meningitidis* serogroup B disease (Sierra et al., 1991) and more are in development for other bacteria (Micoli and Mac Lennan, 2020).

EVs from Gram-negative bacteria contain phospholipids, outer membrane, inner membrane in some cases, cytoplasmic and periplasmic proteins and LPS or lipooligosaccharides (Roier et al., 2015). They may also contain DNA, RNA, ions, metabolites and signaling molecules (Roier et al., 2015; Pathirana and Kaparakis-Liaskos, 2016). In addition, there are reports that EVs can contain small RNAs (sRNAs), which are around 50–200 nt in length and also an important class of post-transcriptional regulators of gene expression (Choi et al., 2017b). Much is already known about the contribution of sRNAs to bacterial fitness in response to different physiological and environmental conditions by distinct mechanisms of regulation (Gripenland et al., 2010; Michaux et al., 2014; Westermann et al., 2016; Hör et al., 2020). Gram-negative species where sRNAs have been found associated with EVs include: *Escherichia coli*, *Pseudomonas aeruginosa*, *Aggregatibacter actinomycetemcomitans*, *Porphyromonas gingivalis*, *Treponema denticola,* and *Salmonella enterica* serovar Typhimurium (reviwed by Badi et al., 2020; Lécrivain and Beckmann, 2020), although the sorting mechanism whereby sRNAs are packaged in EVs is unknown.

The family *Pasteurellaceae* contains several animal and human pathogens, and EVs have proved to be important for cell physiology, and may also be used in immunogenic assays and vaccines to prevent diseases, e.g., *Haemophilus influenzae*, *Pasteurella multocida*, *Mannheimia haemolytica*, *Avibacterium paragallinarum*, *Galibacterium anatis*, *A. actinomycetemcomitans* and *Actinobacillus pleuropneumoniae* (Roier et al., 2013, 2015; Pors et al., 2016; Choi et al., 2017a; Antenucci et al., 2018; Mei et al., 2020). However, with the exception of the human dental pathogen *A. actinomycetemcomitans* (Choi et al., 2017a), there is little data available on sRNAs in EVs of members of the *Pasteurellaceae*. Here, we have characterized EVs produced by *A. pleuropneumoniae*, a Gram-negative coccobacillus, facultative anaerobe and encapsulated member of the *Pasteurellaceae* family, which is the etiological agent of porcine pleuropneumonia, an important disease that causes economic losses worldwide (Pattison et al., 1957; Gottschalk, 2012; Sassu et al., 2018). While there have been studies investigating the sRNAs produced by *A. pleuropneumoniae* (Rossi et al., 2016; Su et al., 2016; da Silva et al., 2022), no information on their association with EVs is available. EVs were prepared from *A. pleuropneumoniae* aerobically and anaerobically grown, in order to best mimic lung infection (Sheehan et al., 2003). The RNAseq analysis led to the discovery of 13 novel sRNAs expressed by the pathogen, some of which have homologues in other *Pasteurellaceae* and or non-*Pasteurellaceae* species.

## 2 Materials and methods

### 2.1 Bacterial strains and growth conditions

The *A. pleuropneumoniae* serovar 8 MIDG2331 strain (Bossé et al., 2016a) was used in this study. The bacterium was routinely grown at 37°C in a 5% CO_2_ atmosphere on brain-heart infusion agar (BHI, BD - 237500) plates supplemented with NAD (10 mg/mL) (Sigma-Aldrich, N0632-5G). For aerobic growth, *A. pleuropneumoniae* was cultivated in BHI-NAD broth at 37°C with agitation (180 rpm). For anaerobic growth, BHI broth was prepared with removal of oxygen and addition of N_2,_ according to Uchino and Ken-Ichiro (2011), with strains being cultivated statically under N_2_ at 37°C.

### 2.2 Growth curves of *Actinobacillus pleuropneumoniae*

For growth curves, under aerobic conditions, *A. pleuropneumoniae* MIDG2331 was cultivated in 50 mL of BHI-NAD in Erlenmeyer flasks incubated at 37°C, for 24 h with agitation (180 rpm). For anaerobic conditions, *A. pleuropneumoniae* MIDG2331 was cultivated in 10 mL BHI-NAD in Hungate tubes incubated at 37°C for 24 h without agitation. Optical density at 600 nm (OD_600_) was measured every hour for the first 12 h, and then at 24 h, using an Ultrospec 10 (GE Healthcare Life Sciences).

### 2.3 Isolation and purification of aerobic- and anaerobic-derived EVs

EVs were derived from aerobic or anaerobic-grown *A. pleuropneumoniae* cultures as described below. For aerobic growth, MIDG2331 was removed from −80°C and cultured on BHI-NAD plates at 37° C and 5% CO_2_, for 24 h. A few colonies were resuspended in 20 mL BHI-NAD broth and incubated at 37°C overnight, with agitation (180 rpm). An aliquot from the overnight culture was transferred to 600 mL of fresh broth, adjusted to an initial OD_600_ of 0.1 and cultivated until late exponential phase. For anaerobic growth, MIDG2331 was cultured on BHI-NAD plates, at 37°C and 5% CO_2_ for 24 h. A few colonies were resuspended in 1 mL of broth and transferred to 10 mL of O_2_-free BHI-NAD in a Hungate tube and incubated overnight, at 37° C. An aliquot of the overnight culture was transferred to 100 mL of a new O_2_-free BHI-NAD and incubated at 37°C, overnight. The culture was transferred to 900 mL of a new O_2_-free BHI-NAD broth (initial OD_600_ ~ 0.1) and cultivated statically under N_2_ at 37°C, until late exponential phase.

EVs from aerobic or anaerobic-grown *A. pleuropneumoniae* were purified as described by Antenucci et al. (2017). Culture supernatants were obtained after centrifugation (20 min at 5,000 *g* at 4°C) and passed through a 0.45 μm filter (Millipore, Billerica, MA, USA). The filtrates were loaded into 1,000 kDa dialysis membranes (Biotech CE Tubing – Spectrum Labs) which were encased in glass columns sealed with transparent film and incubated overnight, at 4°C. The membrane was filled with 600 mL of Phosphate-Buffered Saline (PBS) (300 mL twice) to wash the filtrates and incubated overnight, at 4°C. The filtrates were dialyzed in PBS overnight, at 4°C, with low agitation. The samples were passed through 0.45 μm filters (Cole-Parmer), concentrated with 100 kDa Amicon Ultra-15 Centrifugal Filter Unit (Millipore, Billerica, MA, USA) and stored at −20°C until further analyses were carried out.

### 2.4 Characterization of EVs

#### 2.4.1 Imaging of EVs by transmission electron microscopy

EVs from both aerobic and anaerobic grown bacteria were analyzed by transmission electron microscopy (TEM). Briefly, 10 μL of EVs (0.3 μg protein) were placed on formvar coated grids (Sigma Aldrich), stained with 3% uranyl acetate, and analyzed with a Zeiss EM 109 transmission electron microscope at 80 kV in the Center of Microscopy and Microanalysis (NMM-UFV) facility, at the Universidade Federal de Viçosa.

#### 2.4.2 EV quantification

The samples were analyzed by flow cytometry, and their protein concentrations were determined as described below. For flow cytometry, 20 μL of EVs were treated with 20 μg/mL DNase I (Promega, Promega, Madison, USA). Final volumes of 200 μL of treated EVs were stained with 10 μL of propidium iodide (Live/DeadTM – Thermo Fisher Scientific), following the recommendations of the manufacturer. Finally, EVs were quantified using the BD Accuri C6 flow cytometer (Accuri Cytometers, Belgium) equipped with a 488 nm laser source to promote emissions at FL2 (615–670 nm) and FL3 (> 670 nm). The monoparametric (EVs count vs. propidium iodide fluorescence) and biparametric (EVs count vs. propidium iodide fluorescence vs. SSC) histograms were analyzed using the BD Accuri™ C6 software system.

The EV proteins were quantified by the Bradford reagent (Sigma-Aldrich B6916). Relative quantification was determined by reference to a standard curve obtained using bovine serum albumin (BSA - Sigma-Aldrich - A-4503). A *t*-test was used to determine if the differences in EV production (by protein quantification or flow cytometry) were statistically significant (*p* value <0.05).

#### 2.4.3 EV size distribution

EV size distribution was determined using a dynamic light scattering apparatus (Zetasizer Nano ZS, Malvern Instruments, United Kingdom). The data were analyzed using the Malvern Zetasizer software system version 7.11 to obtain the average hydrodynamic diameter of the particles in solution. The measurements were conducted at 25°C, with three replicate runs of 5 min for each sample, and the average intensity weighted diameter was calculated. Measurements were made on samples containing 30 μg protein of EVs diluted with 1X PBS, pH 7, with a refractive index of 1.332 and viscosity of 0.9043 mPa × s.

### 2.5 EV cargo analysis

#### 2.5.1 Protein profile

Protein profiles of cognate whole-cell extracts, outer membrane protein (OMP) preparations and EVs derived from aerobic and anaerobic-grown *A. pleuropneumoniae* were compared. Whole-cell extracts were prepared from bacterial pellets washed with PBS 1X and transferred to tubes containing Lysing Matrix B beads (MP Biomedicals, CA, USA) and the Precellys 24 tissue homogenizer (Bertin Technologies) used for cell lysis. Lysates were centrifuged (10 min, 16,000 × g, at 4°C), and the supernatants were analyzed. OMP preparations were obtained using “method 1,” as previously described (Thein et al., 2010). Whole-cell extracts, OMP preparations and EVs were dissolved in lysis buffer [50 mM Tris–HCl (pH 6.8); 100 mM dithiothreitol; 2% SDS; 0.1% bromophenol blue; 10% glycerol] and heated for 10 min, at 100°C, and separated by 12% SDS-PAGE, followed by Coomassie blue staining (Sambrook et al., 1989).

#### 2.5.2 Lipid composition

The fatty acid methyl esters (FAMEs) content of EVs (200 μg) obtained from late exponential phase aerobic and anaerobic grown bacteria and 40 μg of cognate pelleted cells (stored at −80° C) were determined. The samples were saponified, derivatized, extracted and washed according to the Sherlock Microbial Identification System (MIS, version 6.2; MIDI, Inc.) and analyzed in a gas chromatograph (Agilent GC 7890 series) coupled with a flame ionization detector (FID) (Agilent, Santa Clara, CA, USA). FAMEs were identified and quantified using the MIDI system (Sherlock Microbial Identification MIDI System of Inc. Newark, Delaware, USA), with experiments conducted in biological triplicate. Peak identification and relative quantification were carried out using the MIDI Sherlock® software system with RTSBA (v. 6.2) library. The Kruskal Wallis test was used to compare the abundance of fatty acids among cells or EVs (*p* values <0.05). The t-test was used to determine whether differences in fatty acid content were statistically significant between cells and EVs (*p-*values <0.05).

LPS was extracted from cognate whole cells (40 μg) and EVs (70 μg) from aerobic and anaerobic-grown bacteria, using hot-aqueous phenol, as described by Davis Jr. and Goldberg (2012). Ten μL of each sample were separated by 16% SDS-PAGE and silver stained (Mortz et al., 2001).

### 2.6 Toxicity of EVs for *Galleria mellonella*

The toxicity of *A. pleuropneumoniae* aerobic and anaerobic-derived EVs against *G. mellonella* (greater wax moth) was determined as previously described (Pereira et al., 2015). In brief, last-instar larvae were injected into the first right pro-leg into the haemocoel, using a 25-gauge microvolume SGE Syringe (26,248 - Sigma-Aldrich). Larvae (10 per experimental replicate) injected with 20 μg EVs from aerobic or anaerobic-grown bacteria were incubated at 37°C and observed for 96 h. Larvae injected with 1X PBS were used as the negative control. The experiments were performed in biological triplicates. Survival curves were plotted using the Kaplan–Meier method (Kleinbaum and Klein, 2012), and statistically significant differences (*p* values <0.05) in survival rate were determined using the log rank test, via the *R* software system, version 2.13.0.

In order to evaluate melanin production, larvae were injected with 3 μg of EVs derived from aerobic and anaerobic grown bacteria, and quantified as described by da Silva et al. (2022). Larvae injected with PBS solution were used as a negative control (the experiments were conducted in biological triplicate). The differences were analyzed using the analysis of variance (ANOVA) followed by the Tuckey test for multiple comparisons, with *p* values <0.05 considered statistically significant.

### 2.7 RNA profile of EVs

#### 2.7.1 RNA extraction

Total RNA was extracted from cognate *A. pleuropneumoniae* MIDG2331 whole cells and EVs from aerobic and anaerobic late exponential-grown bacteria using the miRNeasy kit (Qiagen - Cat. N° 217,084) according to the manufacturer’s instructions. Total RNA was assessed for quantity (Nanodrop 2000c – Thermo Scientific), and for quality by electrophoresis in 0.8% agarose and 12% acrylamide: bis-acrylamide 29:1/8 M urea gels after staining with ethidium bromide solution (0,5 μg/mL).

#### 2.7.2 Sequencing (RNA-seq) and bioinformatic analysis

RNA samples from whole cells and EVs from aerobically grown bacteria were treated with TURBO DNA-free kit™ DNAse (Ambion, Austin, TX), following the manufacturer’s instructions. The small RNA fraction (<200 nt) was obtained from the total RNA samples using the RNeasy MinElute Cleanup kit (Qiagen). The NGS library pool was single-read sequenced on an Illumina NextSeq 500 system using 75 bp read lengths. The output sequences were trimmed with Trimmomatic (Bolger et al., 2014) (parameters: -phred33; ILLUMINACLIP:adapter: 2:30:10; SLIDINGWINDOW: 4:15; LEADING:3; TRAILING:3; MINLEN:30) version 0.36 and the reads were mapped onto the *A. pleuropneumoniae* MIDG2331 genome (GenBank accession number LN908249) using Bowtie2 (Langmead and Salzberg, 2012) version 2.3.4.3 (parameters - local). The resulting bam files were uploaded to NCBIs Short Read Archive (SRA, experiment PRJNA842076). The results were analyzed using the sequence viewer Artemis (Carver et al., 2012). The abundance of RNA categories between EVs and cognate cell data were compared by the *t*-test (*p* values <0.05).

#### 2.7.3 Identification of previously reported *Actinobacillus pleuropneumoniae* sRNAs in EVs

RNAseq data were cross-referenced with previously reported *A. pleuropneumoniae* sRNAs (Rossi et al., 2016; da Silva et al., 2022) (Supplementary Table S1) to determine their presence in EVs. Read counts of previously identified sRNAs in whole cells and EVs were normalized by calculating reads per kilobase million (RPKM). The sRNA abundance was compared using the Kruskal–Wallis test (*p* values <0.05).

#### 2.7.4 Identification of novel sRNAs candidates

The RNAseq mapped data was searched for putative novel sRNA candidates. The approach firstly identified, using Artemis software, increased read regions in RNAseq data from EVs mapped to the annotated genome of MIDG2331. sRNA sequences were then manually delimited and compared to mapped RNAseq data from whole cells. The read coverage of candidate sRNAs was visualized using the integrative genome viewer (Robinson et al., 2023). Normalized read counts (RPKM) were used to compare the abundance of candidate sRNAs in whole cells and EVs. The expression of sRNA candidates was compared using the Kruskal Wallis test (*p* values <0.05).

#### 2.7.5 Validation of sRNA expression in whole cells and presence in the EVs by RT-PCR

sRNAs from whole cells and EVs were extracted and treated with DNase, as described above, prior to cDNA synthesis. The cDNA synthesis was carried out using the High-Capacity cDNA Reverse Transcription Kit (Thermo Fisher Scientific) as recommended by the manufacturer. PCR reactions were performed with 1 U of GoTaq DNA polymerase (Promega) in a final volume of 25 μL of enzyme buffer containing 1.5 mM MgCl_2_, 0.2 mM of each dNTP, and 0.2 μM of each primer. The samples were initially denatured at 95°C, for 2 min, followed by 35 reaction cycles (94°C, for 45 s, 45 s annealing and extension at 72°C, for 30 s) with Tm° dependent on the primers used (primer sequences are listed in Table 1), with a final extension step at 72°C, for 5 min. Whole cell and EV DNase-treated RNA were used as negative controls. Fifty ng of DNA from whole cells was used as the positive control. DNA was extracted from whole cells using the FastDNA SPIN Kit (MP Biomedicals). Whole cells and EVs from aerobically and anaerobically grown bacteria were analyzed for sRNA expression.

**Table 1:** Oligonucleotides used in this work.

**sRNA**	**Primer name**	**Sequence (5′- > 3′)**	**Amplicon length (bp)**	**Reference**
Arrc01	ARRC01_F	TGTTGTGTTTGCATATTGGTCTAGG	122	Rossi et al. (2016)
	ARRC01_R	TGGACGGTTATAAACCAAAAAGGT		
Arrc05	ARRC05_F	CGGTGTGTAAGCGGTCTGAT	103	
	ARRC05_R	GGATACCGAGCTTGTATGCCT		
Arrc07	ARRC07_F	AGGTAGCTGGAGAAGAGCGA	182	
	ARRC07_R	TTCTCCCCTGTCCTTTTGCC		
Arrc08	ARRC08_F	AGAGCAAGCTGATGGTGCTT	160	
	ARRC08_R	CGCTTGCATCGCAAGTAGC		
Arrc11	ARRC11_F	TGTCCAATAAATAGGCTTCCCA	126	
	ARRC11_R	AACTATCCAAATAAAAAGTACGGCT		
Arrc14	ARRC14_F	ACGACTATCTCTTCGACTGCT	103	
	ARRC14_R	GCATCAATGTGCGGGCAAAG		
Arrc17	ARRC17F	TTCTTTCTTGCAAAGAACCCGC	100	
	ARRC17R	ATGCTGATCTTGAAAAGCCCG		
Arrc20	ARRC20_F	GCATTTGACGCTAAAACGGT	128	
	ARRC20_R	AATTAGTGGCTCCTCCTGCG		
Arrc21	ARRC21_F	GACCCTTTAGAAGGCGTTGC	115	
	ARRC21_R	CGCAACGTTAAGGGTCGTTAG		
Rna01	RNA01_F	CTAACTGACAGAATTTATGTAAG	72	da Silva et al. (2022)
	RNA01_R	ACCAAGAAAGCGATGCCG		
Rna02	RNA02_F	ACTTAATAAAAAGTGTTGTG	76	
	RNA02_R	AAGCCCCTCAACTTAGG		
Rna06	RNA06_F	TCATTGGGGTGCTTTACG	55	
	RNA06_R	TCAGATCAGGTTCTACGG		
Rna09	RNA09F	GCTGAACCGACAGCGGAA	103	
	RNA09R	TCCTTAGGTAAGGCGAGCTTC		
Rna10	RNA10_F	CGATTTAATATTCGGGCACTT	95	
	RNA10_R	CAACTCGTATAGGGCGGT		
Rna12	RNA12_F	GAGTGTCAGGTTGTTTT	45	
	RNA12_R	GTCAGAAGCTCCTTTTCA		
EvsRna1	EvsRna1_F	TTGGGCAATTTGTGGTATTTCTT	101	This study
	EvsRna1_R	TGCTTCGTGTTTTTAGCAACG		
EvsRna2	EvsRna2_F	AACTCCCCCTGCTTTGC	55	
	EvsRna2_R	GAAAGCCCCCAACCTTGT		
EvsRna3	EvsRna3_F	ACAGTGATTCATACCTCCAG	66	
	EvsRna3_R	GAAACCCCGTAGAAAACCTAC		
EvsRna4	EvsRna4_F	GCAATTTACCTTCGTTACAGG	97	
	EvsRna4_R	GGGAACAGGGATTTTGTGT		
EvsRna5	EvsRna5_F	AAGCGGTCGGATTTTTAGC	64	
	EvsRna5_R	AGCGGTCAAGATTCATAGC		
EvsRna6	EvsRna6_F	ATGTTTAGCCTTTTGATAAGC	64	
	EvsRna6_R	GGTAGTTTAGTCAGTCGTAG		
EvsRna7	EvsRna7_F	TTCGGACGACACGGAAAG	45	
	EvsRna7_R	GCGAAAAAACAACCGCTTG		
EvsRna8	EvsRna8_F	GGTTAGCAGCCTCCAACT	50	
	EvsRna8_R	ATCCTTGCTTCCACAAGTTG		
EvsRna9	EvsRna9_F	AGGGTTTAGCTATTTCGCCA	59	
	EvsRna9_R	GGCTTAACCTTTCCAGTTTCAG		
EvsRna10	EvsRna10_F	GACCGTTCGTGAATTGTCG	152	
	EvsRna10_R	CGAGAGTAAATTGGGGCGT		
EvsRna11	EvsRna11_F	CAGAAAAAGCCCGCAAATTG	69	
	EvsRna11_R	TCTGCACCTTAATCCGTTAGAG		
EvsRna12	EvsRna12_F	CTTGTGGGAGAGGGACAG	101	
	EvsRna12_R	GCAGAGAGAGGGGAATTTGC		
EvsRna13	EvsRna13_F	ATACCTGCCGTGTAGTTGG	194	
	EvsRna13_R	ATTAACGGTTGGTCAGGTTG		
EvsRna14	EvsRna14_F	TTTGATCTGTTACTGG	68	
	EvsRna14_R	TGTTACGCCCTCTCTC		
EvsRna15	EvsRna15_F	TGCTTCTTGTAATATTAACGT	53	
	EvsRna15_R	ACTGACGGTTGCATATCAA		
5S	APP5SF	GCGATGCCCTACTCTCACAT	100	Rossi et al. (2016)
	APP5SR	GAGTGCTGTGGCTCTACCTG		

#### 2.7.6 Structural characterization of the sRNA candidates

sRNA candidates that were confirmed by RT-PCR were evaluated for their novelty by searching the Rfam database (version 14.7). The secondary structure and free energy (∆G) of the sRNAs confirmed by RT-PCR were predicted using RNAfold (Lorenz et al., 2011). Putative promoters of the sRNA candidates were predicted using BProm^1^ by analyzing up to 100 bp upstream of their predicted starts. In order to investigate the putative association of the sRNA candidates with the RNA chaperone Hfq, the putative Hfq-binding sequence was manually inspected based on the sequence “GGGUUUUUUU” (Holmqvist et al., 2016). Subsequently, the search for homologues was performed using BLASTn and the PATRIC database with a 70% cutoff for coverage and identity. The presence of the novel sRNAs candidates among bacterial species was visualized in a network developed using Cytoscape (Shannon et al., 2003).

#### 2.7.7 Localization of sRNAs in *Actinobacillus pleuropneumoniae* EVs

The internal localization of sRNAs associated with EVs was determined by comparison of treatment with or without RNase prior to RNA extraction, as described by Koeppen et al. (2016). Briefly, EVs were treated using 0.5 μg/mL of RNase A (Qiagen) for 30 min, at 37°C, and washed with PBS in a 100 kDa Amicon Ultra Centrifugal Filter Unit (Millipore, Billerica, MA, USA) to remove the RNAse. sRNAs were extracted, and cDNA was obtained as described above. Only the sRNAs confirmed in the total RNA of EVs were tested for their presence inside EVs, as determined by protection from RNase degradation.

## 3 Results

### 3.1 Analysis of EVs

#### 3.1.1 EV production by *Actinobacillus pleuropneumoniae* under different growth conditions

**Figure 1:**
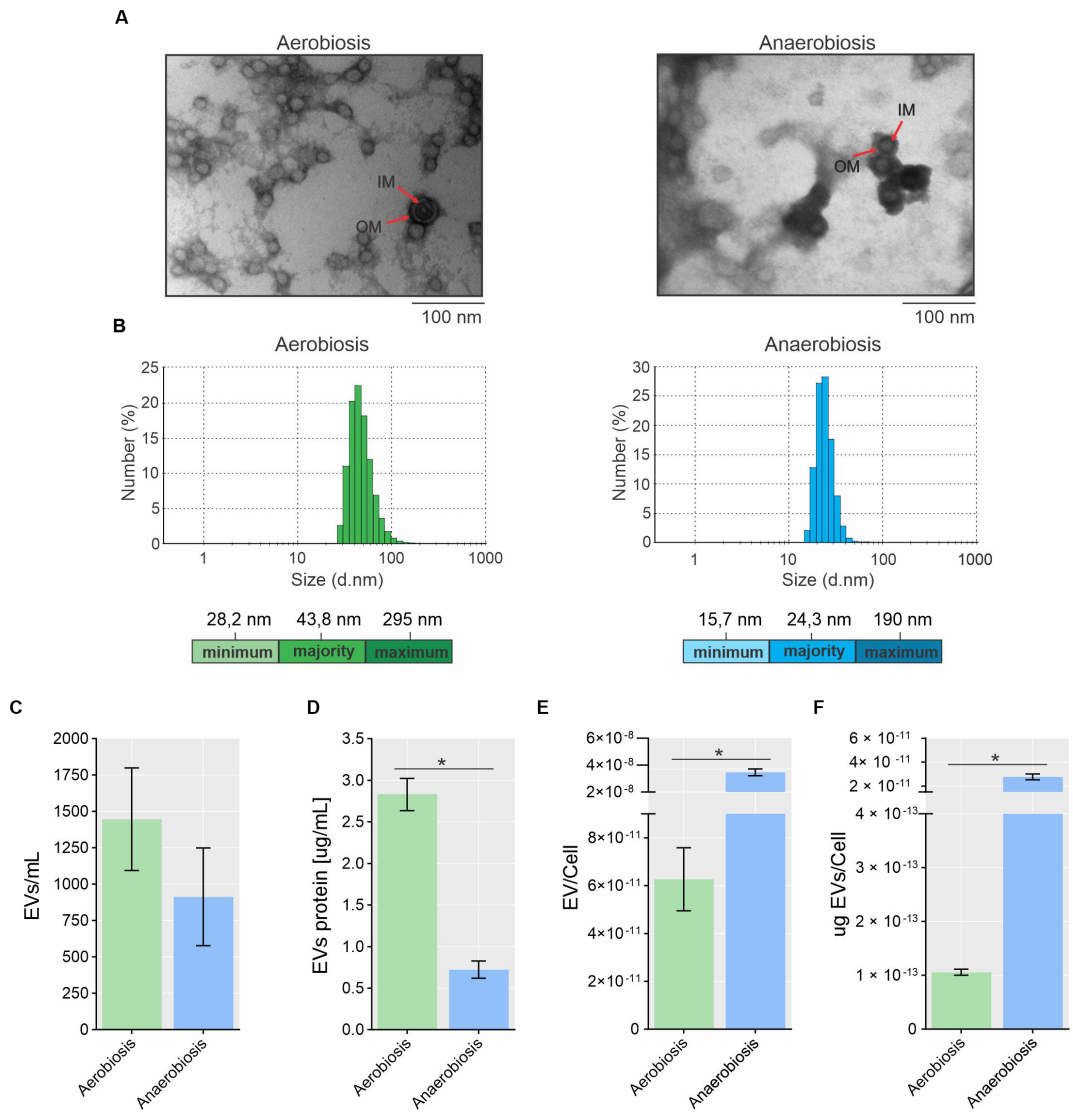
Morphology, size and production of EVs from *A. pleuropneumoniae*. **(A)** Transmission electron microscopy (TEM) of EVs from aerobic (left) and anaerobically (right) grown bacteria. In both cases, EVs with two membrane layers encasing electron dense “cytoplasmic” material are indicated by arrows. OM and IM correspond to the outer membrane and inner membrane, respectively. **(B)** EV size and dispersity measured by dynamic light scattering (DLS). The minimum, most present and maximum size of the EVs are shown at the bottom of each Figure. **(C)** Quantification of EV numbers by flow cytometry. **(D)** EV protein quantification. Standardization of EV production quantified by flow cytometry **(E)** or by protein **(F)** per cell. Significant differences between aerobically and anaerobically EV production are shown by “*”, as calculated by *t*-tests (*p*-values <0.05).

The EVs from *A. pleuropneumoniae* were harvested from the late exponential phase for both aerobic (7 h, OD_600_ ~ 2.75 and ~ 2.36 × 10^13^ CFU/mL) and anaerobic (8 h, OD_600_ ~ 0.8 and ~ 2.64 × 10^10^ CFU/mL) growth (Supplementary Figure S1) and used for further characterization and analyses. The EVs were obtained from the supernatant of the same culture in which we evaluated the protein, lipid and RNA content of the cells, so, they are directly comparable. EVs produced by aerobically and anaerobically grown *A. pleuropneumoniae* were analyzed by TEM and had their integrity confirmed. The EVs presented a circular morphology, with one or two membranes and an electron dense content for EVs with two membranes (Figure 1A). Most small EVs exhibited only one membrane, although it is possible to observe a big EV with only one membrane from anaerobiosis. Most bigger EVs presented two membranes with electron dense content (Figure 1A). TEMs of MIDG2331 EVs were similar in appearance to those previously reported for the same strain isolated by the same technique (Antenucci et al., 2017; Zhu et al., 2022). EVs produced by *A. pleuropneumoniae* under different culture conditions differed in size and dispersity. The most prevalent diameter of EVs from aerobically grown bacteria was approximately 44 nm (~23%), with sizes ranging between 28 nm and 295 nm (Figure 1B). In contrast, the most prevalent EV diameter of anaerobically-derived EVs was approximately 24 nm (~28%), with variation from ~16 to 190 nm.

Flow cytometry quantification revealed that EV production was higher in aerobic compared to anaerobic culture, with ~1,446 and ~ 913 EVs per mL, respectively (Figure 1C), although this was not statistically significant (*p* = 0.365). By protein quantification using the Bradford reagent, *A. pleuropneumoniae* grown aerobically and anaerobically produced 2.57 and 0.72 μg of EVs per mL of culture, respectively: A statistically significant difference (*p* = 0.0002) (Figure 1D). The ratio of EVs (quantified by flow cytometry or protein amount)/CFU demonstrated that significantly more EVs were produced anaerobically than aerobically (*p* = 0.001 for both methods) (Figures 1E,F).

#### 3.1.2 EV protein and lipid profiles

**Figure 2:**
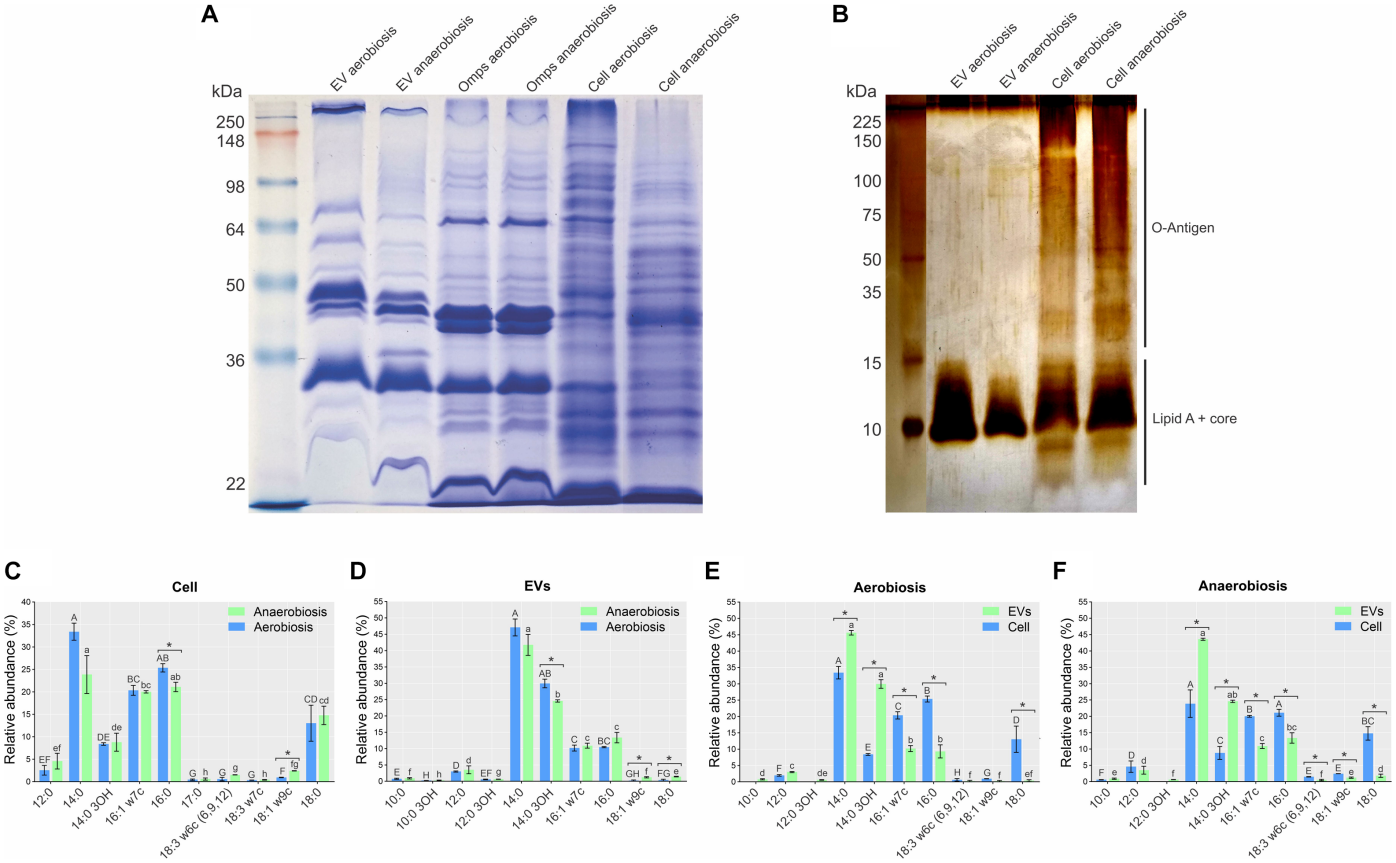
Whole cell, EV, and outer membrane protein (OMP) and lipopolysaccharide (LPS) profiles from aerobically and anaerobically grown *A. pleuropneumoniae.* **(A)** Protein profiles of EVs, OMP preparations and total whole cell extracts. Ladder: See Blue Plus Prestained (Invitrogen). **(B)** LPS profiles of EVs and their cognate whole cells. Ladder: Broad Range Protein Molecular Weight Marker (Promega). **(C–F)** Relative abundance and fatty acid profiles of EVs and their cognate whole cells derived from aerobic and anaerobically grown bacteria. Comparisons of fatty acid composition: **(C)** between whole cells; **(D)** between EVs; **(E)** between cells and EV from aerobiosis; **(F)** between cells and EVs from anaerobiosis. Significant differences between averages of the same fatty acid are represented by “*” after comparisons, as determined by *t*-tests (*p*-values <0.05). Differences found by Kruskal Wallis tests of fatty acid abundance are represented by capital letters (“A” to “H”) for aerobiosis condition and lowercase letters (“a” to “h”) for anaerobiosis in **(C,D)**. Differences found by the Kruskal–Wallis test of fatty acid abundance are represented by capital letters (“A” to “H”) for cells; and lowercase letters (“a” to “f”), for EVs in **(E,F)**.

The protein profiles of EVs from both growth conditions were similar, but there were subtle differences (Figure 2A). The EV protein profiles were different from their whole cell counterparts (Figure 2A). Unsurprisingly, the EV protein profiles were most similar to those of the OMP preparations (Figure 2A).

The LPS profiles of EVs from both growth conditions were different from that of whole cells. The LPS profile of whole cells was the smooth type (containing O-antigen, core and lipid A), while that of the EVs was the rough type (containing core and lipid A) (Figure 2B). Minor differences were observed in the LPS profiles of whole cells grown aerobically and anaerobically, but not between the EVs from both growth conditions (Figure 2B).

There was only a small difference in fatty acid composition between *A. pleuropneumoniae* whole cells and EVs grown aerobically and anaerobically. The most abundant fatty acids in whole cells grown in both conditions were myristic acid (14:0) (*p-*value <0.05), followed by palmitic acid (16:0), palmitoleic acid (16:1 w7c), stearic acid (18:0) and 3-hydroxitetradecanoic acid (14:0 3OH) (Figure 2C). In EVs, 14:0, 14:0 3OH, 16:0 and 16:1 w7c were the most abundant fatty acids (*p-*value <0.05) (Figure 2C). By comparing the cells, significant differences were observed only for 16:0 and 18:1 w9c (*p* = 0.08 and *p* = 0.00001) (Figure 2C). For the EV comparisons, significant differences were observed for 14: 0 3OH, 18:1 w9c and 18:0 (*p* = 0.011, 0.014 and 0.03) (Figure 2D). Comparing EVs and cells, for both aerobiosis and anaerobiosis, 14:0 (*p* = 0.005 for aerobiosis and *p* = 0.008 for anaerobiosis) and 14:0 3OH (*p* = 0.001 for aerobiosis and *p* = 0.003 for anaerobiosis) had higher abundance in EVs. Conversely, 16:1 w7c (*p* = 0.001 for aerobiosis and *p* = 0.001 for anaerobiosis), 16:0 (*p* = 0.006 for aerobiosis and *p* = 0.002 for anaerobiosis) and 18:0 (*p* = 0.017 for aerobiosis and p = 0.005 for anaerobiosis) had higher abundance in cells (Figures 2E,F). EVs also exhibited a higher amount of saturated fatty acids, compared to their cognate cells (~77% for cells and ~ 89% for EVs).

### 3.2 *Actinobacillus pleuropneumoniae* EVs are toxic for *Galleria mellonella*

After investigating the protein and LPS profiles of EVs, we evaluated the toxicity potential of the EVs for the wax moth *G. mellonella*. The results revealed that growth conditions used to produce EVs did not affect the survival of the larvae. At 96 h, only ~50% of the larvae were dead when injected with EVs (Supplementary Figure 2A). No death of larvae injected with PBS occurred during the 96 h.

Melanization quantification showed a slight difference only at 24 h in larvae infected with EVs from aerobiosis (*p* = 0.019). However, no significant difference was observed until 96 h between larvae infected with EVs from aerobiosis or anaerobiosis, also revealing melanization reduction during the experiment (Supplementary Figures 2B,C). Also, we observed a significant difference between the melanin measurement from larvae infected with EVs from aerobiosis and anaerobiosis conditions and the control (larvae injected with PBS) only at 24 h (Supplementary Figure 2B).

### 3.3 RNA cargo from *Actinobacillus pleuropneumoniae* EVs

**Figure 3:**
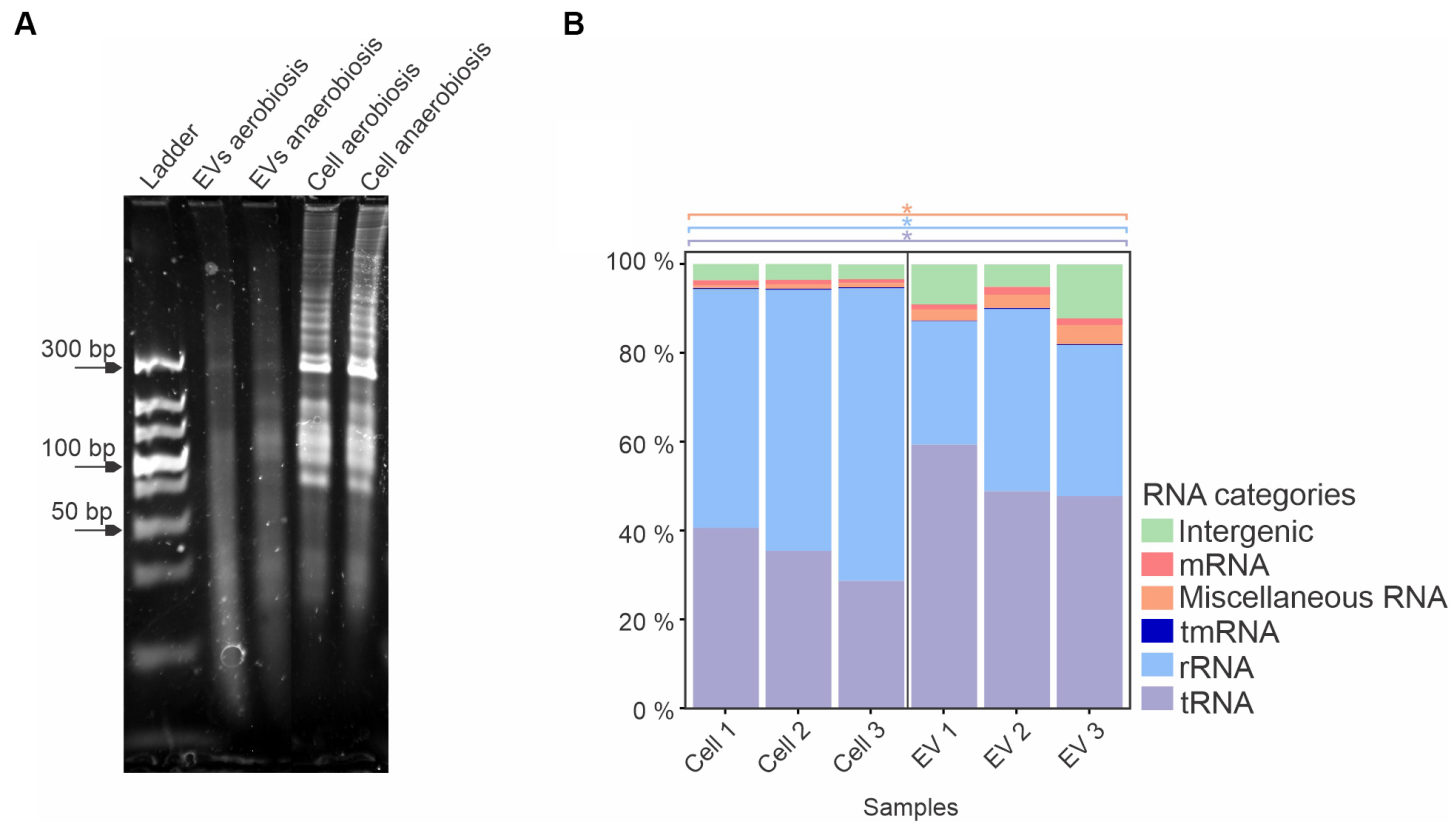
*A. pleuropneumoniae* EVs have a diverse RNA content. **(A)** 10% denaturing urea gel of EV and whole cell RNA from aerobic and anaerobically grown bacteria. Ladder: Ultra Low Range DNA Ladder (Thermo Fisher Scientific). **(B)** Total RNA composition of EVs from aerobic growth. Significant differences between EVs and whole cell groups are represented by “*” for each category of RNA (indicated by different colors), as calculated using the *t*-test (*p*-values <0.05).

When qualitatively analyzed by denaturing polyacrylamide urea gel electrophoresis and ethidium bromide staining, a similar pattern of RNAs was observed in EVs from both growth conditions, with slight differences. Total sRNAs from whole cells also presented a similar profile, with a predominant band at 300 bp, while there was a variety of sRNA sizes from whole cells compared to EVs (Figure 3A). Since no major differences were observed between the EVs produced by *A. pleuropneumoniae* under aerobic and anaerobic conditions, the identification and characterization of total and EV-associated RNA were conducted only with samples from aerobically grown bacteria. After RNA sequencing from aerobiosis and mapping to the *A. pleuropneumoniae* MIDG2331 from triplicate experiments, there were 13,462,604, 9,166,775 and 9,766,708 reads from total RNA and 134,070, 686,819 and 152,393 reads from the EVs mapped to the *A. pleuropneumoniae* MIDG2331 genome. The RNAseq analysis of *A. pleuropneumoniae* EVs demonstrated that they contained diverse classes of RNAs, including mRNAs, miscellaneous RNA (MiscRNAs), tmRNA, rRNA, tRNA and intergenic RNAs (Figure 3B). Moreover, tRNAs and MiscRNAs were more abundant in EVs than whole cells (*p-*values <0.05) (Figure 3B). RNAseq confirmed the results obtained by denaturing polyacrylamide gel electrophoresis, i.e., that EVs contain sRNAs.

**Figure 4:**
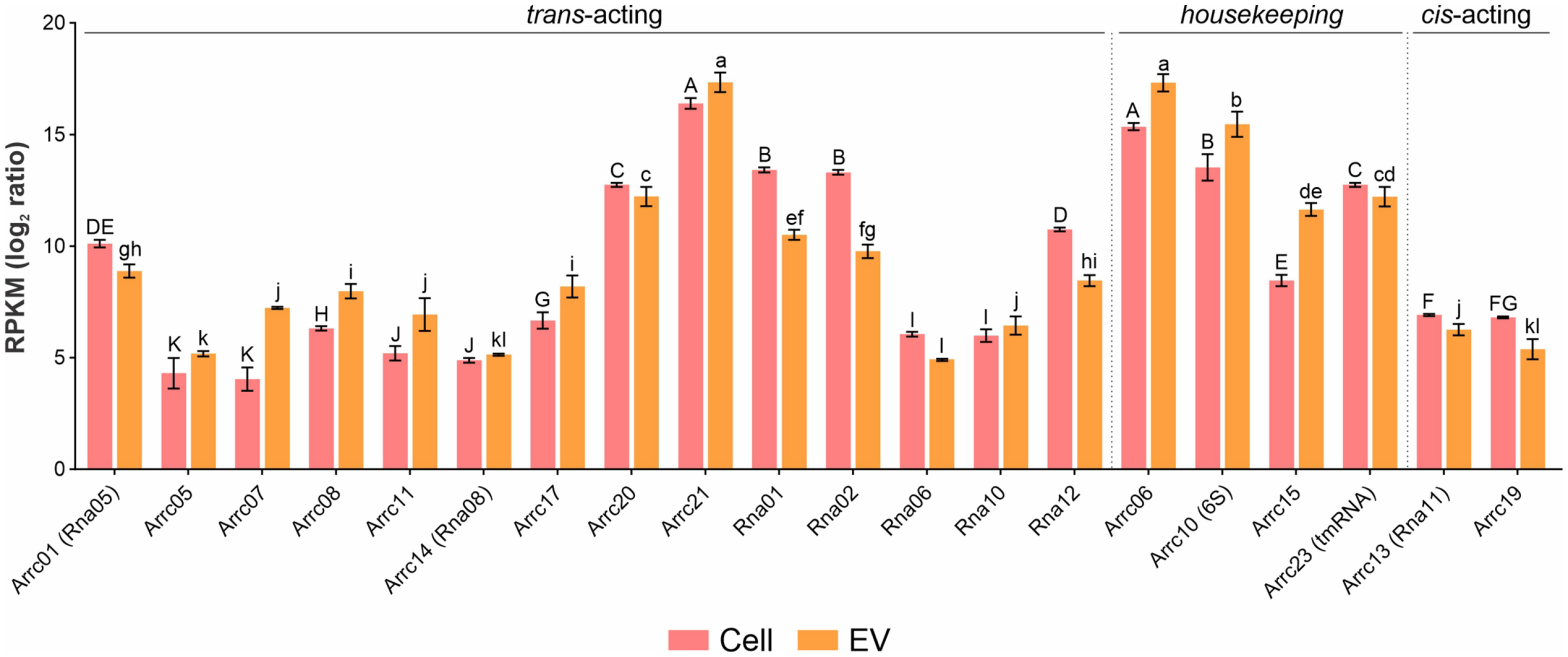
sRNA abundance in *A. pleuropneumoniae* EVs. Comparison of the relative abundance of trans-coding sRNAs in EVs and whole cells. sRNA abundance is represented by RPKM. sRNA categories are indicated above the bars. Differences found by Kruskal–Wallis tests of sRNA abundance are represented by capital letters (“A” to “K”) for cells and lowercase letters (“a” to “l”) for EVs.

Among the housekeeping sRNAs present in EVs, tRNAs encoding all amino acids were found, among which asparagine, glutamate and tyrosine were the most abundant (*p-*values <0.05) (Supplementary Figure S3). Also, several tRNAs, e.g., tryptophan and tyrosine, were enriched in EVs compared to whole cells (Supplementary Figure S4A). Twenty previously reported *A. pleuropneumoniae* sRNAs (Rossi et al., 2016; da Silva et al., 2022) (Supplementary Table S1) were found in EVs, of which Arrc21 and Arrc06 were the most abundant (*p-*values <0.05) (Figure 4). Moreover, some sRNAs were more abundant in EVs than their respective producing cells, as observed for Arrc05, 06, 07, 08, 10 (6S), 11, 15, 17, 21 and Rna10 (Supplementary Figure S4B).

#### 3.3.1 Novel sRNA candidates found in *Actinobacillus pleuropneumoniae* EVs

**Figure 5:**
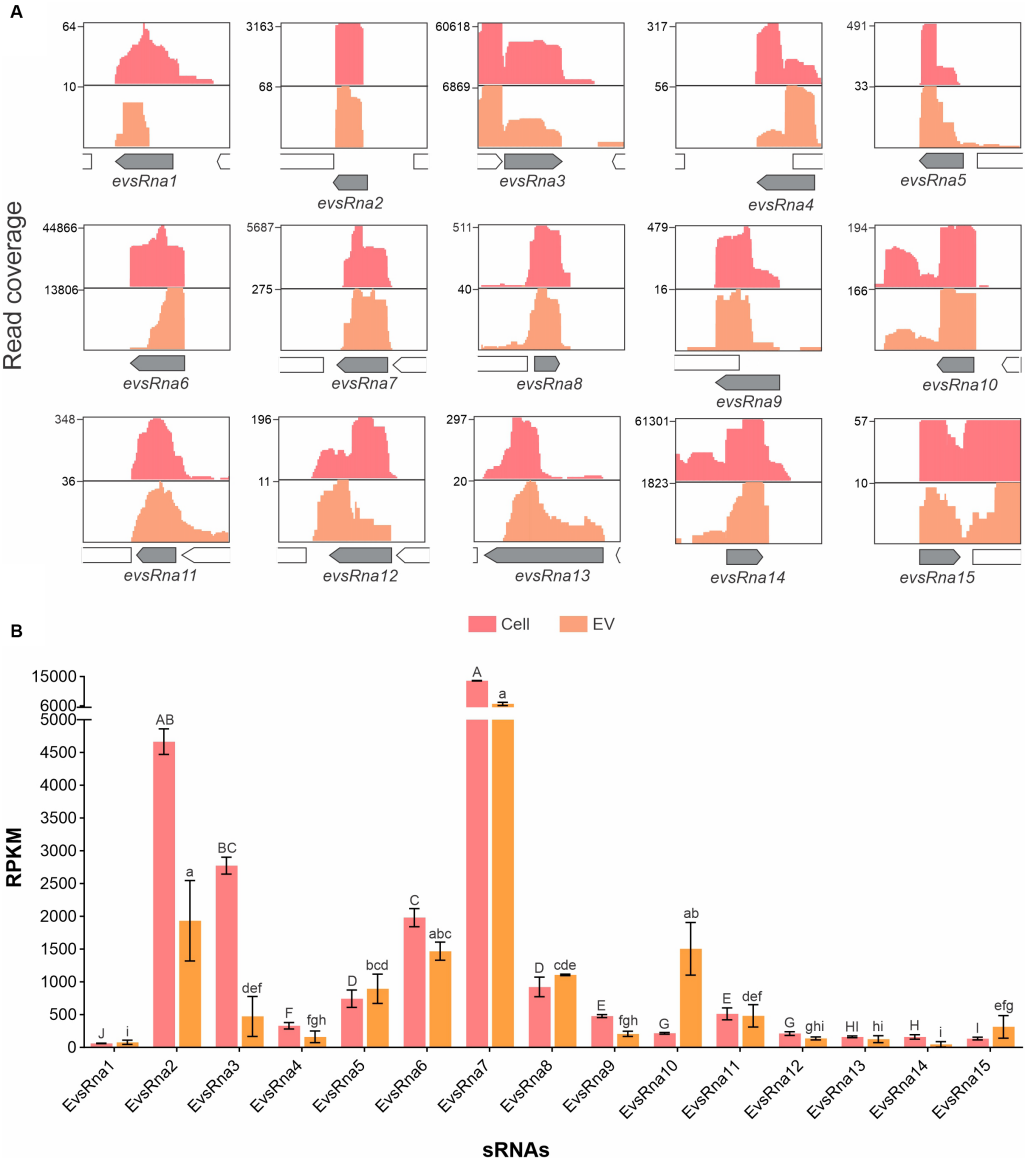
Gene organization and relative abundance of novel sRNA candidates from EVs and cognate whole cells of *A. pleuropneumoniae*. **(A)** Increased RNA-seq reads in intergenic regions from EVs and whole cells mapped to the MIDG2331 genome. The mapping was carried out using the integrative genome viewer. **(B)** Comparison of the abundance of *trans*-coding sRNAs between whole cells and EVs. The sRNA abundance is represented by RPKM. Differences found by Kruskal–Wallis tests of sRNAs abundance are represented by capital letters (“A” to “J”) for cells, and lowercase letters (“a” to “i”) for EVs.

Detailed analysis of RNA-seq results with manual inspection identified 15 possible new *A. pleuropneumoniae* sRNA candidates, named as EvsRna1 to 15. All of them are localized in intergenic regions of the *A. pleuropneumoniae* MIDG 2331 genome (Table 2 and Figure 5A). The size of the novel sRNA candidates ranged from 53 to 248 nt, which is consistent with the EV sRNA profile (Figure 3A). Putative −10 and −35 regions of σ^70^ promotors were found for all the novel sRNA candidates except for EvsRna3 and EvsRna5 (Table 2). By visual inspection, a putative Hfq RNA chaperone binding site was observed in 11 sRNA candidates, i.e., EvsRna1, 2, 3, 4, 6, 7, 10, 11, 12, 13, and 15 (Table 2). All candidates were considered as novel sRNAs, since they did not match with any family in the Rfam database. EvsRna7 was the most abundant in the EVs (by RPKM comparison) (*p-*value <0.05) (Figure 5B). The candidates EvsRna5, 10 and 15 were more abundant in EVs compared to their cognate whole cell samples (Supplementary Figure S4C).

**Table 2:** Novel sRNA candidates associated with EVs from *A. pleuropneumoniae.*

**Candidate**	**Position**	**Size** **(bp)**	**Strand**	**Putative** **−35**	**Putative** **−10**	**Putative** **Hfq- binding site**	**Upstream gene**	**Downstream gene**	Homologue in *Pasteurellaceae*/other families*
EvsRna1	217434..217566	133	−	TTGAAT	TGCGATAAT	UCGUUUUUUU	*mcrA*	*aroK*	Yes/No
EvsRna2	324793..324857	65	−	TTGATC	CTGTAAAAT	UUCUUUUUUA	MIDG2331_00301	MIDG2331_00302	Yes/Yes
EvsRna3	351412..351530	119	+	−	−	GGUUUCUUUU	tRNA-Asn	*rumA*	Yes/No
EvsRna4	755602..755733	132	−	TTGACT	GGCTAGAAT	AGGGAUUUUU	MIDG2331_00698	*mtfA*	Yes/No
EvsRna5	848973..849047	75	−	−	−	No	MIDG2331_00786	*rec2*	Yes/No
EvsRna6	896360..896467	108	−	TTGCAA	TAGAAT	UUUUUAAUUU	MIDG2331_01325	MIDG2331_01324	Yes/No
EvsRna7	1039104..1039175	72	−	TTGCCG	CTAAATAAT	UCAACUUUUU	*iscU*	*iscA1*	Yes/No
EvsRna8	1322264..1322316	53	+	TTTCGT	GACTATCCT	No	*rpsF*	*purD*	Yes/No
EvsRna9	1432325..1432455	131	−	TTGAAA	TAAAAT	No	MIDG2331_01325	MIDG2331_01324	No/No
EvsRna10	1558709..1558900	192	−	ATGGCG	TATACT	CGGAUUUUUA	*topB2_2*	MIDG2331_01469	Yes/No
EvsRna11	1695309..1695397	89	−	TATACT	TGCTAAGG	CAGAUUUUUU	MIDG2331_01606	MIDG2331_01605	Yes/Yes
EvsRna12	1892321..1892453	133	−	GTGACC	ATAAAAATA	GCAAUUUUCU	*ureA*	*ureB*	Yes/No
EvsRna13	2021328..2021575	248	−	TTGAAA	ATAGTA	CGGUUUUUUU	*gloA2*	*sfsA*	Yes/No
EvsRna14	2035311..2035394	84	+	TTTACA	TAAGAT	No	MIDG2331_01951	tRNA-Asn	Yes/No
EvsRna15	2292439..2292513	75	+	TTGCAA	TATAAT	AGUUUUUUGA	*comF*	*rsmC*	Yes/No

^*^Homologs were considered by BLASTn against NCBI and PATRIC databases with a cutoff of 70% for coverage and identity.

The expression of the 15 *trans-acting* sRNAs previously reported in *A. pleuropneumoniae* (Supplementary Table S1) was confirmed in aerobically-grown bacteria by RT-PCR. Out of the sRNAs whose expression was confirmed in whole cells, Rna02 and Rna09 were the only ones that were not found in EVs (Table 3). With the exception of EvsRna3 and EvsRna5, the expression of novel sRNA candidates was confirmed in whole cells and detected in the EVs. EvsRna7, EvsRna11 and EvsRna12 were not found inside aerobically-derived EVs (Table 3). Anaerobically, only Rna02, EvsRna3 and EvsRna5 expression was not confirmed by RT-PCR. In addition, anaerobically, Arrc08, Rna10 and EvsRna11 were the only sRNAs whose expression was found in whole cells but not detected in the EVs (Table 3).

**Table 3:** Expression of *A. pleuropneumoniae trans* sRNAs in whole cells and presence in cognate EVs as determined by RT-PCR.

**sRNA**	**Expressed in aerobic cells**	**Confirmed in aerobic EVs**	Confirmed inside aerobic EVs *	**Expressed in anaerobic cells**	**Confirmed in anaerobic EVs**
Arrc01	Yes	Yes	Yes	Yes	Yes
Arrc05	Yes	Yes	Yes	Yes	Yes
Arrc07	Yes	Yes	Yes	Yes	Yes
Arrc08	Yes	Yes	Yes	Yes	No
Arrc11	Yes	Yes	Yes	Yes	Yes
Arrc14	Yes	Yes	Yes	Yes	Yes
Arrc17	Yes	Yes	Yes	Yes	Yes
Arrc20	Yes	Yes	Yes	Yes	Yes
Arrc21	Yes	Yes	Yes	Yes	Yes
Rna01	Yes	Yes	Yes	Yes	No
Rna02	Yes	No	NA**	No	No
Rna06	Yes	Yes	Yes	Yes	Yes
Rna09	Yes	No	NA**	Yes	Yes
Rna10	Yes	Yes	Yes	Yes	No
Rna12	Yes	Yes	Yes	Yes	Yes
EvsRna1	Yes	Yes	Yes	Yes	Yes
EvsRna2	Yes	Yes	Yes	Yes	Yes
EvsRna3	No	No	NA**	No	No
EvsRna4	Yes	Yes	Yes	Yes	Yes
EvsRna5	No	No	NA***	No	No
EvsRna6	Yes	Yes	Yes	Yes	Yes
EvsRna7	Yes	Yes	No	Yes	Yes
EvsRna8	Yes	Yes	Yes	Yes	Yes
EvsRna9	Yes	Yes	Yes	Yes	Yes
EvsRna10	Yes	Yes	Yes	Yes	Yes
EvsRna11	Yes	Yes	No	Ye	No
EvsRna12	Yes	Yes	No	Yes	Yes
EvsRna13	Yes	Yes	Yes	Yes	Yes
EvsRna14	Yes	Yes	Yes	Yes	Yes
EvsRna15	Yes	Yes	Yes	Yes	Yes

^*^Only for EVs produced aerobically. ^**^NA: not investigated inside EVs because they were not found in the total RNA of the vesicles.

#### 3.3.2 Characterization of novel sRNA candidates found in *Actinobacillus pleuropneumoniae* EVs

**Figure 6:**
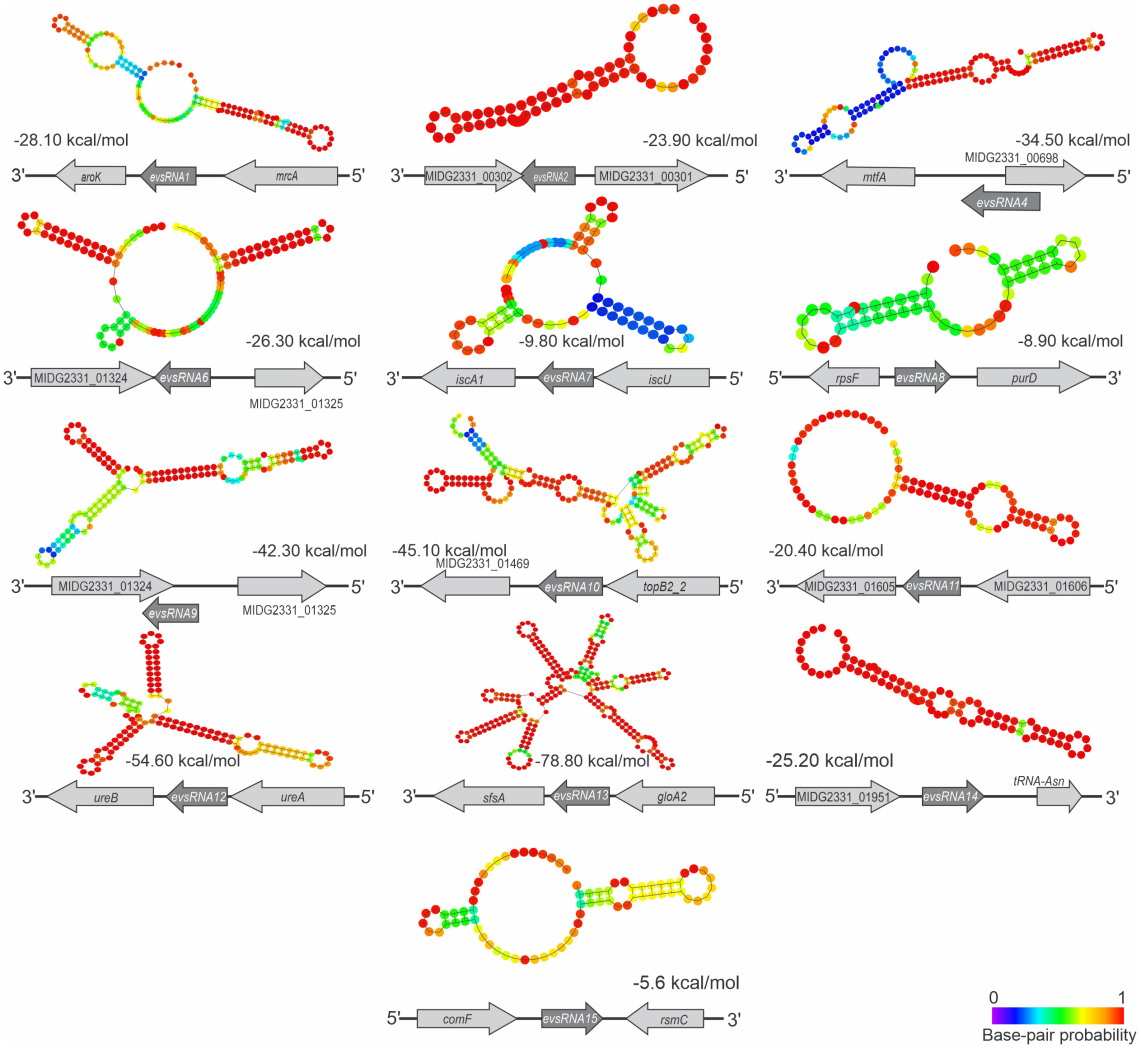
Structure and context of novel *A. pleuropneumoniae* sRNA candidates. The sRNA structures were predicted using RNAfold (Lorenz et al., 2011). Context and strand of the candidates are represented below each sRNA structure. The calculated free energy (ΔG) of sRNA structures, provided by RNAfold, is shown below each structure. The base pair probability is presented in the heatmap at the bottom-right.

The gene organization in *A. pleuropneumoniae* and the predicted secondary structures of the novel sRNAs with confirmed gene expression are shown in Figure 6. Database searching identified homologues of some sRNAs in a wide variety of *Pasteurellaceae* species, e.g., EvsRna8, and *Pasteurellaceae* and non-*Pasteurellaceae* species, e.g., EvsRna2 and EvsRna11 (Supplementary Figure S5), which included *Neisseria* spp. and members of the *Enterobacteriaceae* family, respectively (Supplementary Figure S5). Moreover, homologues of EvsRna2 and EvsRna11 were also found in partial sequences of Caudoviricetes. The analysis of the gene context of the homologues of EvsRna2, EvsRna8, EvsRna11, EvsRna12, and EvsRna14 in other species found that the flanking genes were different, particularly in non*-Actinobacillus* species. The evaluation of the GC content of sRNA homologues found that, for all candidates, except EvsRna8 and EvsRna11, the GC content is similar to their counterparts in the MIDG2331 genome, with approximately ±3% variation.

## 4 Discussion

EVs are nanoparticles known to be important in mediating bacterial-bacterial and host-bacterial interactions (Gill et al., 2019). In Gram-negative bacteria, they comprise proteins, LPS, and can also contain nucleic acid (Gill et al., 2019). Here, we have isolated EVs from *A. pleuropneumoniae*, an economically important pig pathogen. Specifically, we have compared protein, fatty acid, and LPS profiles of EVs and cognate whole cells from *A. pleuropneumoniae* grown aerobically and anaerobically. The present study also sought to identify, for the first time to our knowledge, the sRNA cargo of *A. pleuropneumoniae* EVs, an under-investigated area.

*A. pleuropneumoniae* EVs were isolated from aerobic and anaerobically-grown cultures, reflecting the respiratory states typical of laboratory settings and lung infection, respectively. TEMs of EVs from both growth conditions presented double-membrane structures containing electron dense material similar to EV structures reported for MIDG2331 prepared by a similar method (Antenucci et al., 2017). However, the size of the EVs produced was different. In this study, the most prevalent size of aerobically-derived EVs was 43.8 nm (range 28.2–295 nm), while the most prevalent size of EVs under the same condition was 73 nm (range 56–212 nm) in the study described by Antenucci et al. (2017), which may be due to the different methods used to measure EV size.

Protein quantification and flow cytometry indicated that the total amount of EVs produced was greater in aerobic compared to anaerobic growth. However, after the normalization of EV production by CFUs, anaerobic *A. pleuropneumoniae* were found to produce more EVs than those grown aerobically. Previous studies have reported that the availability of oxygen affects EV production, as described for *N. meningitidis* and *P. aeruginosa* (Schertzer et al., 2010; Gerritzen et al., 2018; Toyofuku et al., 2019).

The protein profiles of EVs were similar to those from cognate OMP preparations, although no Coomassie blue-stained bands >98 kDa were found. Antenucci et al. (2019) and Zhu et al. (2022) also found other proteins than OMPs in MIDG2331 EVs, as adjudged by protein profile. Additionally, despite the significant differences in abundance, the fatty acid profiles of EVs were similar to whole cells. EVs from both growth conditions contained more saturated fatty acids than their cognate whole cells. Saturated fatty acids decrease membrane fluidity (Hąc-Wydro and Wydro, 2007), a reduction of the membrane fluidity was associated with an increased EV production in *P. aeruginosa* (Mashburn-Warren et al., 2008). Fatty acids are constituents of LPS. The EVs produced by *A. pleuropneumoniae* contained rough type LPS, while the smooth type was present in whole cells. Fatty acids containing 14, 16, and 12 carbons were predominant in the EVs, which is consistent with their presence in lipid A, as described for a range of bacterial species (Steimle et al., 2016; Kawahara, 2021). According to Jefferies and Khalid (2020), the type of LPS in EVs may be associated with the morphology and uptake by host cells. EVs containing the rough type may lose the sphericity when in contact with host cell surface, and the uptake may be slower and less efficient by the host cell. Also, EVs containing rough LPS induces a different host immune response when compared to the EVs containing smooth LPS, as demonstrated by Avila-Calderón et al. (2012) in a study with *Brucella melitensis*. Taken together, the results indicate that the EVs, unsurprisingly, are predominantly derived from outer membrane constituents.

EVs were toxic for *G. mellonella* (Supplementary Figure S2), with 50% survival at 96 h and rapid melanization. Melanization was higher and more rapid than previously reported for *G. mellonella* larvae infected with live *A. pleuropneumoniae* (Pereira et al., 2015; da Silva et al., 2022). The melanization is part of the humoral response of *G. mellonella* and can be described as the synthesis and deposition of melanin to encapsulate pathogens at the wound (Tsai et al., 2016). The EVs are rich in LPS, which is a microbe associated molecular pattern (MAMP), which elicits an immune response in *G. mellonella* (Parusel et al., 2017) and may explain the high and rapid melanization of the larvae infected with the EVs.

The virulence of *A. pleuropneumoniae* is multifactorial and complex, commonly involving LPS, capsule, siderophores and the RTX toxins ApxI, ApxII, and ApxIII, which possess cytotoxic and/or hemolytic activity (Frey, 2011). Virulence-associated proteins, including Apx toxins, have been found in *A. pleuropneumoniae* EVs (Negrete-Abascal et al., 2000; Antenucci et al., 2017, 2018, 2019; Zhu et al., 2022), but the *A. pleuropneumoniae* factors that initiate the *G. mellonella* melanization cascade remain unclear. Apx toxins (ApxI and ApxII are produced by MIDG2331) are unlikely mediators, as culture supernatants did not induce the melanization or killing of *G. mellonella* larvae (Pereira et al., 2015), and Apx toxins have a tropism for porcine cells (Kuhnert et al., 2003). Culture supernatants would also have contained EVs and their constituent LPS, which in other Gram-negatives has proved to be a potent inducer of the *G. mellonella* immune response, while triggering an early-melanization response (Wu et al., 2015; Elizalde-Bielsa et al., 2023). Further work is required to determine if a specific factor or a combination of *A. pleuropneumoniae* virulence factors present in EVs can induce melanization. EVs from Gram-negative bacteria have been classified into outer membrane vesicles (OMVs), formed by the blebbing of the outer membrane, and outer-inner membrane vesicles (OIMVs), typically formed by explosive cell lysis (Toyofuku et al., 2019). OMVs are characterized by the presence of a single membrane bilayer, and OIMVs, by the possession of two membrane bilayers derived from the outer and inner membranes sandwiching a peptidoglycan layer. TEMs identified EVs with OMV and OIMV characteristics. The comparative protein profiles suggest that the EVs were primarily composed of OMPs, which is consistent with the proteomic data on EVs from the same strain prepared by the same method as this study (Antenucci et al., 2019; Zhu et al., 2022). However, OMVs do not typically contain inner membrane and/or cytoplasmic content such as nucleic acids (Toyofuku et al., 2019), as we have found in this study. The TEMs and presence of nucleic acid (sRNAs) suggest that our EV preparations contained both OMVs and OIMVs. While we did not evaluate the proportion of each EV type produced under the different growth conditions, it is known that the ratio can vary (Toyofuku et al., 2019).

In the present work, we have demonstrated that bacterial EVs contain nucleic acids and, in particular, an sRNA pool. sRNAs are known for their role in the virulence of diverse bacterial species. Most of the information is related to the action of these molecules on cell regulation during the infection process. Thus, there is still very little information about the role of these RNAs outside the cell and those transported by EVs in the infection process. Our study, which reports the presence of sRNAs packaged in EVs produced by the porcine pathogen *A. pleuropneumoniae,* is one of the few works that address the *Pasteurellaceae*.

We have studied the RNA content of EVs produced by *A. pleuropneumoniae* aerobically and anaerobically grow. The RNAseq analysis identified a diverse profile of RNAs in the EVs, and tRNAs and rRNAs were the most abundant. Most tRNAs were enriched in EVs compared to whole cells. The high abundance of tRNAs and rRNAs in whole cells is expected due to their functions, but it is not known whether there are specific mechanisms that result in enrichment in EVs. Koeppen et al. (2016) found a fragment of a tRNA, named sRNA52320, in *P. aeruginosa,* which is associated with the modulation of the immune response of human airway cells. Diallo et al. (2022) also found a fragment of tRNA, named as Ile-tRF-5X, classified as a very small RNA (vsRNA), in *E. coli*, which is associated with the promotion of the MAP3K4 expression in human HCT116 cells. Pérez-Cruz et al. (2021) also found a high amount of tRNAs in EVs produced by *P. aeruginosa*. Moreover, Ghosal et al. (2015) identified a higher amount of tRNAs in EVs produced by *E. coli*. In this study, MiscRNAs, tmRNAs and intergenic sequences were found in *A. pleuropneumoniae* EVs in proportions different from those described for *E. coli* and *S.* Typhimurium (Ghosal et al., 2015; Malabirade et al., 2018). Similar to the diverse profile of RNAs found in the EVs produced by *A. pleuropneumoniae*, Langlete et al. (2019) also demonstrated that EVs produced by *Vibrio cholerae* carry diverse RNAs, some being enriched in the EVs. Further work is required to determine whether EV RNA content and composition are intrinsic to each species and the extent to which it is growth condition-dependent.

We found diverse RNAs associated with the EVs, including 14 *trans* sRNAs previously reported as being expressed by *A. pleuropneumoniae* (Rossi et al., 2016; da Silva et al., 2022). Moreover, 15 novel *A. pleuropneumoniae* sRNA candidates were identified, including13 confirmed by RT-PCR. All newly reported sRNAs were expressed from intergenic regions in the genome, including EvsRna10 present in the integrative conjugative element ICE*Apl1* (Bossé et al., 2016b). Also, all the newly confirmed sRNAs presented putative promotors for σ^70^. However, all but EvsRna4 and 7 had stable predicted secondary structures. Some homologues were found in diverse species, as observed for EvsRna8 and 11, which are widely found in *Pasteurellaceae* and non-*Pasteurellaceae* species. In general, sRNA homologues have GC content similar to *A. pleuropneumoniae,* but their gene context was different. Hfq-dependent Rna01 is one of the sRNAs found in the EVs, and was previously reported in association with *A. pleuropneumoniae* OMP regulation and stress responses (da Silva et al., 2022). Since we cannot exclude the possibility that the other sRNAs transported in the EVs are associated with gene regulation of targets in *A. pleuropneumoniae*, we searched for the presence of Hfq binding sites. Most candidates presented a putative Hfq binding sequence. However, the action of these candidates in the *A. pleuropneumoniae* must be further investigated. By means of *in silico* and *in vitro* investigation, we observed that most of the sRNAs confirmed in whole cells were present in EVs produced during aerobic growth.

Choi et al. (2017a) had already reported the presence of small RNAs in EVs produced by the periodontal pathogen *A. actinomycetemcomitans*. Despite reports of the identification of msRNAs (sRNAs with microRNA size) differing in size from those sRNAs reported here, they revealed the potential of msRNAs to modulate the immune response of host cells by EVs delivery.

Out of the known and newly identified sRNAs, only three (Arrc08, Rna10, EvsRna11) were expressed in whole cells, but not found in the EVs produced during anaerobic growth (Table 3). Thus, unsurprisingly, the sRNA content of the EVs produced by *A. pleuropneumoniae* is growth condition-dependent. Some sRNAs, e.g., EvsRna5, 10, and 15, were enriched in EVs compared to their cognate whole cells. Similarly, sRNA enrichment was found in EVs produced by *P. aeruginosa* (Resch et al., 2016) and *V. cholerae* (Langlete et al., 2019). It has been suggested that the contents of different types of EVs are inherently linked to biogenic processes, although the mechanisms remain obscure (Toyofuku et al., 2019).

The majority of sRNAs were packaged within EVs, as determined by RT-PCR and for the protection from RNase degradation. However, some were not protected from RNase degradation and were assumed to be attached to the outer surface of the EVs, as described for *P. aeruginosa* (Koeppen et al., 2016). Whether there are specific attachment mechanisms to the surface of EV and the role of extracellular-bound sRNAs in virulence remains to be determined.

In summary, we have identified that known and novel sRNAs are present in EVs of *A. pleuropneumoniae* grown aerobically and anaerobically. In some cases, there was enrichment of sRNAs within EVs compared to their cognate whole cells, while others were externally-associated but not present within EVs. Homologues of some sRNAs were found in other *Pasteurellaceae* and also non-*Pasteurellaceae* species. However, the role of EVs and sRNAs in bacteria-bacteria and bacterial-host interactive biology remains to be determined.

## Data availability statement

The RNA sequencing presented in this study was uploaded to NCBIs Short Read Archive (SRA, experiment PRJNA842076).

## Author contributions

GS: Conceptualization, Data curation, Formal analysis, Investigation, Methodology, Validation, Visualization, Writing – original draft, Writing – review & editing. JR: Formal analysis, Investigation, Methodology, Writing – review & editing. PF: Formal analysis, Investigation, Methodology, Writing – review & editing. AC: Formal analysis, Methodology, Writing – review & editing. EB: Formal analysis, Methodology, Writing – original draft. WC: Formal analysis, Methodology, Writing – original draft. HM: Methodology, Writing – original draft. YL: Methodology, Writing – original draft. JB: Conceptualization, Data curation, Formal analysis, Investigation, Methodology, Supervision, Visualization, Writing – original draft. PL: Conceptualization, Funding acquisition, Project administration, Resources, Supervision, Visualization, Writing – review & editing. DB: Conceptualization, Funding acquisition, Methodology, Project administration, Resources, Supervision, Visualization, Writing – original draft, Writing – review & editing.

## Footnotes

^1^ http://www.softberry.com
