# Supplementary material for: Identification of novel small RNAs in extracellular vesicles produced by Actinobacillus pleuropneumoniae
Source: Front Microbiol. 2023 Nov 23;14:1291930. doi: 10.3389/fmicb.2023.1291930 (PMC10708910; doi:10.3389/fmicb.2023.1291930)
Supplement: Supplementary file 1 [file Data_Sheet_1.docx]

Supplementary Material

**Supplementary Table 1**. List of sRNAs previously confirmed in *A. pleuropneumoniae.*

| **Name** | **Type** | **Position** | **Reference** |
| --- | --- | --- | --- |
| Arrc01/Rna05 | *trans* – acting | 149354..149556 | Rossi et al. (2016)¹ |
| Arrc05 | *trans* – acting | 760650..760819 |  |
| Arrc06 | *housekeeping* | 896755..896868 |  |
| Arrc07 | *trans* – acting | 1279204..1279310 |  |
| Arrc08 | *trans* – acting | 1997375..1997557 |  |
| Arrc10/6S | *housekeeping* | 131770..131947 |  |
| Arrc11 | *trans* – acting | 211986..212067 |  |
| Arrc13 | *cis*-acting | 440496..440646 |  |
| Arrc14/Rna08 | *trans* – acting | 451950..452112 |  |
| Arrc15 | *housekeeping* | 563241..563629 |  |
| Arrc17 | *trans* – acting | 2064961..2065080 |  |
| Arrc19 | *cis*-acting | 2308407..2308602 |  |
| Arrc20 | *trans* – acting | 2118118..2118483 |  |
| Arrc21 | *trans* – acting | 2100216..2100291 |  |
| Arrc23/tmRNA | *housekeeping* | 2118118..2118483 |  |
| Rna01 | *trans* – acting | 738604..738689 | da Silva et al. (2022)² |
| Rna02 | *trans* – acting | 662472..662552 |  |
| Rna06 | *trans* – acting | 195041..195191 |  |
| Rna09 | *trans* – acting | 1869469..1869534 |  |
| Rna10 | *trans* – acting | 1996019..1996142 |  |
| Rna12 | *trans* – acting | 2292548..2292720 |  |

¹ Rossi, C. C., Bossé, J. T., Li, Y., Witney, A. A., Gould, K. A., Langford, P. R., & Bazzolli, D. M. S. (2016). A computational strategy for the search of regulatory small RNAs in *Actinobacillus pleuropneumoniae*. RNA (New York, N.Y.), 22(9), 1373–1385. https://doi.org/10.1261/rna.055129.115

² da Silva, G. C., Rossi, C. C., Rosa, J. N., Sanches, N. M., Cardoso, D. L., Li, Y., Witney, A. A., Gould, K. A., Fontes, P. P., Callaghan, A. J., Bossé, J. T., Langford, P. R., & Bazzolli, D. M. S. (2022). Identification of small RNAs associated with RNA chaperone Hfq reveals a new stress response regulator in *Actinobacillus pleuropneumoniae*. Frontiers in Microbiology, 13:1017278. https://doi.org/10.3389/fmicb.2022.1017278


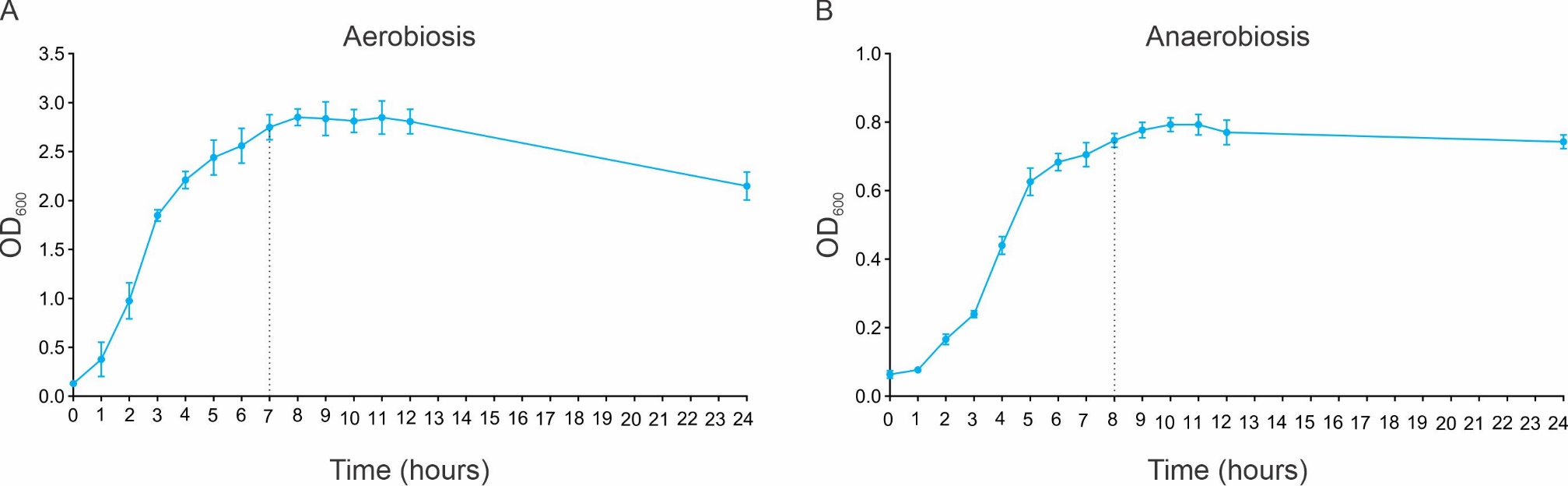


Supplementary Figure 1 - Growth of *A. pleuropneumoniae* MIDG2331. (A) Growth curve of *A. pleuropneumoniae* in aerobic condition. (B) Growth curve of *A. pleuropneumoniae* in anaerobic condition. The dotted line in each graph represents the time that the incubation ceased (late exponential phase), and the supernatant was used for EVs purification. For both conditions, the EVs were obtained from the supernatant of the same culture in which we evaluated the protein, lipid and RNA content of the cells, so, they are directly comparable.


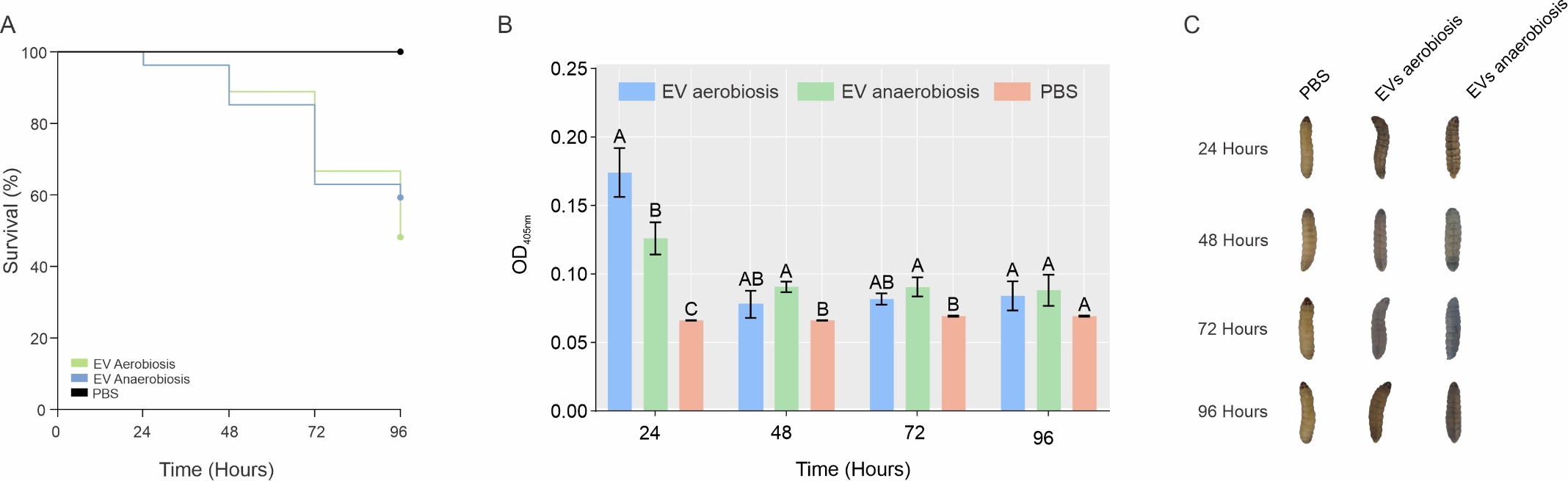


**Supplementary Figure 2. Toxicity of EVs for the greater wax moth *G. mellonella*.** (A) *G. mellonella* killing assay. (B) Optical density (OD_405nm_) and melanization of larval haemolymph post-infection. (C) Visual observation of larval melanization through the course of the experiment. Means with different letters (A, B or C) are significantly different by Tuckey’s test (p<0.05).


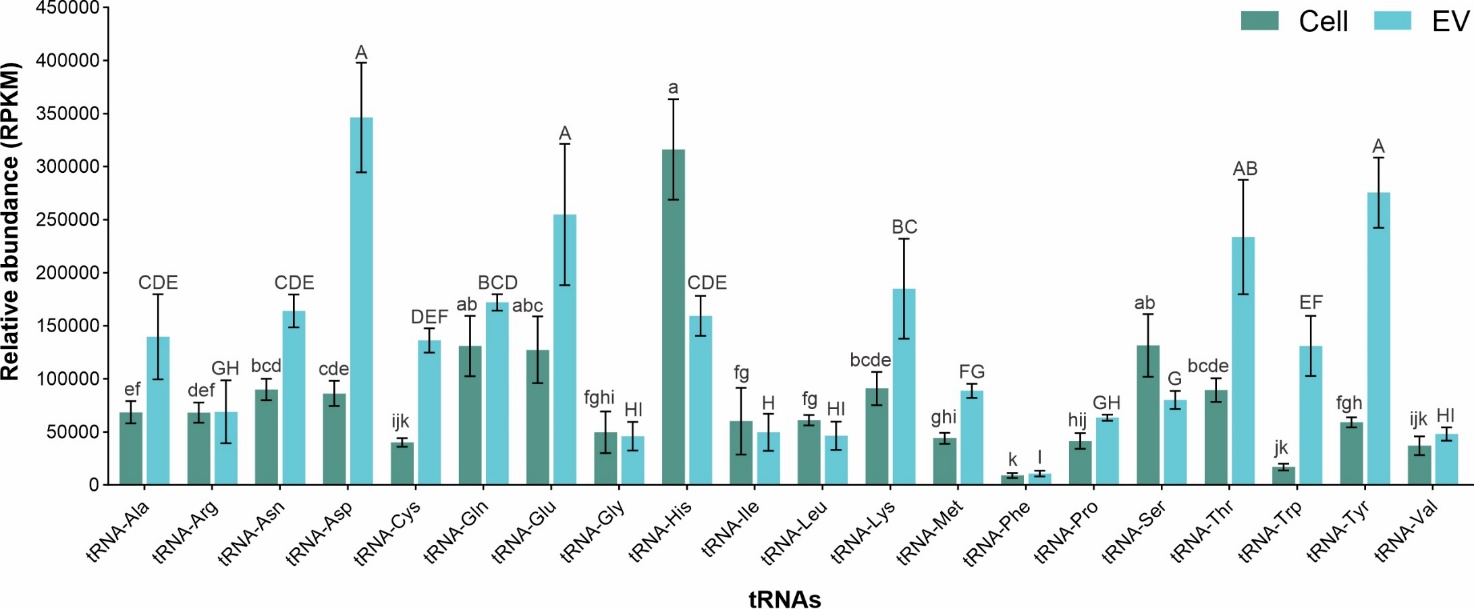


**Supplementary Figure 3. tRNAs are abundant in *A. pleuropneumoniae* EVs.** Comparison of the relative abundance of tRNAs in the EVs and whole cells. tRNAs abundance is represented by RPKM. Differences found by Kruskal Wallis test of the sRNAs abundance are represented by capital letters (“A” to “I”) for EVs and lowercase letters (“a” to “k”) for Cells.


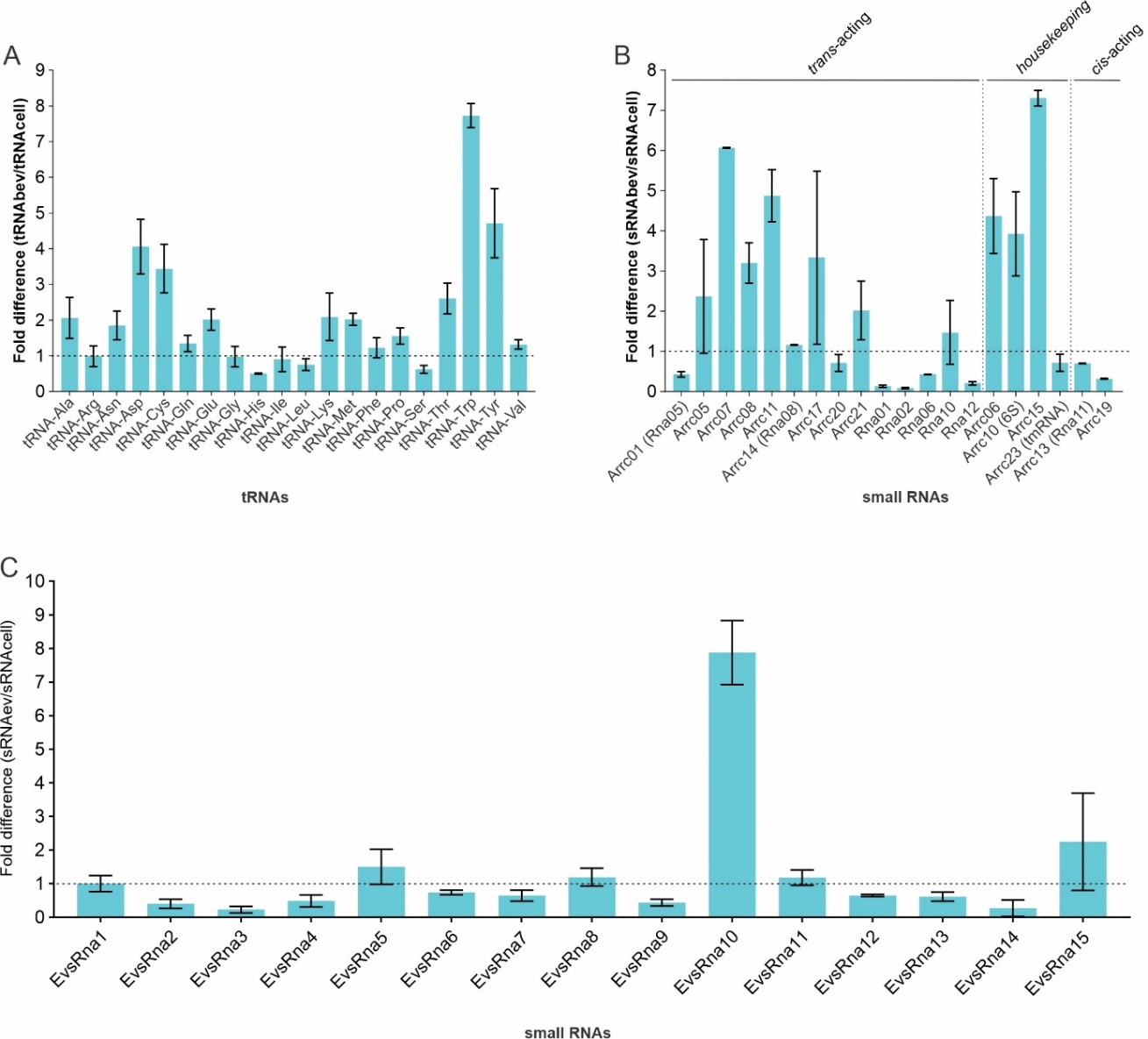


**Supplementary Figure 4. Some sRNAs are enriched in EVs produced by *A. pleuropneumoniae***. Fold difference (ratio of EV/whole cell RNAs) of (A) tRNAs and (B) sRNAs previously reported in *A. pleuropneumoniae*, and (C) novel sRNAs candidates identified in this work (C). The bars above the dotted lines represent the RNAs enriched in the EVs compared to whole cells. The bars below the dotted lines represent RNAs enriched in whole cells compared to EVs. In B, sRNA classes are shown above the bars.


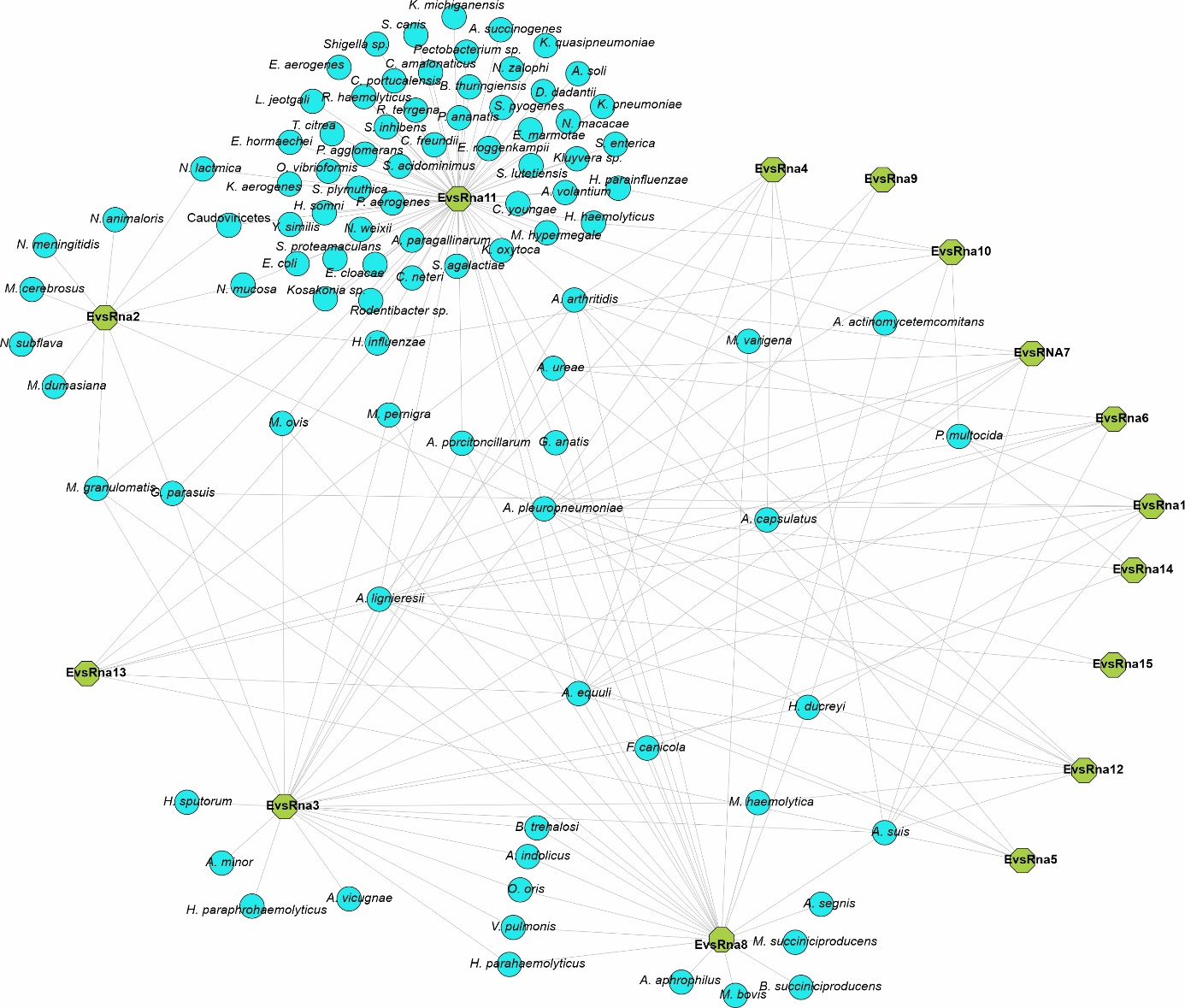


**Supplementary Figure 5. Presence of homologues of novel *A. pleuropneumoniae* sRNAs identified in this work among other bacterial species.** The novel *A. pleuropneumoniae* sRNA candidates are represented by green nodes and those from other species by the light blue nodes. The network was generated using Cytoscape, considering as homologues, sequences with a 70% cutoff.
